# Evaluation of treatment-associated eye toxicity after irradiation in childhood and adolescence—results from the Registry of the Evaluation of Side Effects after Radiotherapy in Childhood and Adolescence (RiSK)

**DOI:** 10.1007/s00066-021-01793-2

**Published:** 2021-06-07

**Authors:** Fenja Albrecht, Heidi Wolters, Yvonne Ziert, Beate Timmermann, Rolf-Dieter Kortmann, Christiane Matuschek, Christian Rübe, Carmen Martini, Hans Christiansen, Hans Theodor Eich, Normann Willich, Diana Steinmann

**Affiliations:** 1 Department of Radiotherapy, Medical School Hannover, Hannover, Germany; 2 Department of Radiotherapy, University Hospital of Münster, Münster, Germany; 3 Institute of biometrics, Medical School Hannover, Hannover, Germany; 4 Center for Proton Radiation Therapy, Paul-Scherrer-Institute, Villigen, Switzerland; 5 Department of Particle Therapy, West German Proton Therapy Centre Essen (WPE), West German Cancer Center (WTZ), German Cancer Consortium (DKTK), University Hospital Essen, Essen, Germany; 6 Department of Radiotherapy, University of Leipzig, Leipzig, Germany; 7 Department of Radiation Oncology, Heinrich Heine University Hospital of Düsseldorf, Düsseldorf, Germany; 8 Department of Radiotherapy and Radiation Oncology, Saarland University, Homburg/Saar, Germany; 9 Department of Radiotherapy, University Hospital of Freiburg, Freiburg, Germany

**Keywords:** Radiation, Eye, Late toxicity, Side effects, Childhood

## Abstract

**Purpose:**

The aim of the study is to evaluate treatment-related acute and late eye toxicity associated with radiation therapy in childhood and adolescence as correlated with RT (radiotherapy) doses.

**Methods:**

From 2001 to 2016, a total of 1725 children and adolescents undergoing radiation therapy were prospectively documented in the Registry of the Evaluation of Side Effects after Radiotherapy in Childhood and Adolescence (RiSK). The RTOG/EORTC criteria were used to classify ocular acute and late effects. Uni- and multivariate analyses were carried out to evaluate the impact of patient age, pre-existing impairments, and radiation dose on ocular toxicity.

**Results:**

Of all documented patients, 593 received dose to the eye and formed the basis of this analysis. In 435 patients, information on acute reaction was available and graded 1, 2, 3, and 4 in 49, 17, 0, and 2 patients, respectively. Information on late toxicity was available in 268 patients and graded 1, 2, 3, and 4 in 15, 11, 11, and 5 patients, respectively. The acute toxicity rate was significantly higher in children who received a maximum dose > 50 Gy to the eye (*p* < 0.001) and who had a pre-existing eye impairment (*p* < 0.001 in multivariate analysis). The development of late toxicity was significantly higher for patients experiencing acute toxicity and having received a radiation dose > 50 Gy.

**Conclusion:**

Acute and late toxicity both correlate with high radiation dose to the eye (> 50 Gy) and acute toxicity additionally with pre-existing eye impairments.

## Introduction

Approximately one third of pediatric oncology patients need radiation therapy during the treatment of their malignant disease [1, 2]. The survival rates have significantly improved during the past years, with the 5‑year-survival rate nowadays being over 80% [3]. Therefore, evaluation of treatment-related acute or late adverse effects is of major importance [4]. The acute (defined from start until 90 days after radiation therapy) and late (occurring after more than 90 days after radiation therapy) effects can be documented organ specifically by the RTOG/EORTC criteria [5, 6].

The eye, specifically the lens, is a sensitive structure and may develop relevant acute and late disorders, including cataract and retinopathy, after radiation therapy in children and adolescents [7–10].

To standardize the documentation of acute and late effects after radiation therapy in pediatric patients the Registry of the Evaluation of Side Effects after Radiotherapy in Childhood and Adolescence (RiSK) was founded in 2001 by the German Working Group of Paediatric Radio-Oncology (APRO) chaired by N. Willich, Münster. Financial support was provided by the *Deutsche Kinderkrebsstiftung*. In addition to the basic data of the radiation therapy, the acute and late effects to relevant organs at risk were documented according EORTC/RTOG [6] for each patient. Evidence of a correlation between radiation dose, radiation volume, and occurrence of acute and late effects in several organs, such as liver, lung, and thyroid gland has already been published using the RiSK database [11–13].

The aim of this study was to evaluate treatment-associated acute and late effects in the eye after radiation therapy in childhood and adolescence under consideration of the radiation dose–response relationship and further influencing factors.

## Materials and methods

The Registry of the Evaluation of Side Effects after Radiotherapy in Childhood and Adolescence includes altogether 1725 children and adolescents in 62 German centers, who underwent radiation therapy between 2001 and 2016. A standardized documentation of the applied radiotherapy including doses to organs at risk and adverse events based on RTOG/EORTC criteria of the patients was carried out and recorded in the database [1, 2, 6]. The classification of acute and late effects of the eye is displayed in Table 1. The Ethics Committee of the University Hospital Münster approved this project before starting documentation.

**Table 1:** Maximum acute/late eye toxicity i(RTOG/EORTG criteria) [6]

	Maximum acute eye toxicity	Late toxicity on the eyes
Grade 0	No change	None
Grade 1	Mild conjunctivitis with or without scleral injection/increased tearing	Asymptomatic cataract Minor corneal ulceration or keratitis
Grade 2	Moderate conjunctivitis with or without keratitis requiring steroids and/or antibiotics/dry eye requiring artificial tears/iritis with photophobia	Symptomatic cataract Moderate corneal ulceration; minor retinopathy or glaucoma
Grade 3	Severe keratitis with corneal ulceration/objective decrease in visual acuity or in visual fields/acute glaucoma/panophthalmitis	Severe keratitis; severe retinopathy or detachment Severe glaucoma
Grade 4	Loss of vision (unilateral or bilateral)	Panophthalmitis/Blindness

Of the 1725 registered children and adolescents, all patients having received any dose to the eye were included in this evaluation (*n* = 593). Also patients with total body irradiation (TBI) were included, as well as patients with an eye disorder already existing before radiation therapy. Patients with re-irradiation close to the eye and those with missing data were excluded.

In the registry, the radiation doses to the right eye and left eye were documented separately. For evaluation, we used the maximum dose (right or left eye), determined as Dmax, for the further statistical analysis (Table 2). To better analyze the influence of the irradiation dose on ocular toxicity, a classification based on the Dmax value was performed (referring to Whelan et al.), as listed in Table 2; [7]. Late toxicity had been documented in most patients at each follow-up (median 2.5 follow-ups [range 1–11]) and the maximum level of late toxicity reported was used for the analysis. Also included in the analysis were the three most common diagnoses ALL, medulloblastoma, and rhabdomyosarcoma (Table 2).

**Table 2:** Baseline patient characteristics

	All patients (%)	Patients with documented acute toxicity (%)	Patients with documented late toxicity (%)
*Total*	*593*	*435 (73.4)*	*268 (45.2)*
*Sex*			
Male	338 (57.0)	244 (56.1)	151(56.3)
Female	255 (43.0)	191 (43.9)	117 (43.7)
*Age in years* (median)	8.48 years (range 0.77–20.1 years)	8.68 years (range 0.77–20.1 years)	8.62 years (range 0.86–20.1 years)
*Age*			
0–4 years	149 (25.1)	112 (25.7)	65 (24.3)
5–9 years	185 (31.2)	135 (31.0)	84 (31.3)
10–14 years	155 (26.1)	119 (27.4)	70 (26.1)
15–20 years	89 (15.0)	64 (14.7)	43 (16.0)
*Most frequent diagnosis*			
ALL	114 (19.2)	58 (13.3)	45 (16.8)
Rhabdomyosarcoma	86 (14.5)	84 (19.3)	58 (21.6)
Medulloblastoma	63 (10.6)	47 (10.8)	28 (10.4)
Astrocytic tumor (WHO grade I–IV)	60 (10.1)	47 (10.8)	19 (7.1)
Sarcoma	27 (4.6)	23 (5.3)	14 (5.2)
Lymphoma	13 (2.2)	10 (2.3)	4 (1.5)
AML	27 (4.6)	10 (2.3)	13 (4.9)
Ependymoma	29 (4.9)	25 (5.7)	12 (4.5)
Craniopharyngioma	23 (3.9)	19 (4.4)	11 (4.1)
Other	151 (25.5)	112 (25.7)	64 (23.9)
*Preexisting eye impairment*	145 (24.5)	121 (27.8)	73 (27.2)
*Median eye dose in Gy*	46.20 Gy (range 0.0–69.0 Gy)	51.10 Gy (range 0.0–69.0 Gy)	48.05 Gy (range 0.0–68.4 Gy)
*Classification Dmax*			
Group 1 (0–2.0 Gy)	21 (3.5)	12 (2.8)	7 (2.6)
Group 2 (> 2.0–5.0 Gy)	8 (1.3)	4 (0.9)	4 (1.5)
Group 3 (> 5.0–12.0 Gy)	124 (20.9)	55 (12.6)	52 (19.4)
Group 4 (> 12.0–20.0 Gy)	38 (6.4)	26 (6.0)	12 (4.5)
Group 5 (> 20.0–40.0 Gy)	68 (11.5)	54 (12.4)	32 (11.9)
Group 6 (> 40.0–50.0 Gy)	64 (10.8)	53 (12.2)	27 (10.1)
Group 7 (> 50.0 Gy)	270 (45.5)	228 (52.4)	131 (48.9)
*Total body irradiation*	95 (16.0)	34 (7.8)	31 (11.6)
*Chemotherapy*			
Any CTx	495 (83.5)	359 (82.5)	208 (77.6)
Before	435 (87.9)	309 (71.0)	191 (71.3)
Simultaneous	260 (52.5)	196 (45.1)	106 (39.6)
After	249 (50.3)	186 (42.8)	106 (39.6)
Median time for last follow up (months)	25.27 months	24.38 months	27.04 months

Continuous variables are reported as median (min–max), categorial variables are reported as absolute frequencies and relative frequencies (%)

*ALL* acute lymphatic leukemia, *AML* acute myelogenous leukemia, *CTx* chemotherapy

### Statistical analysis

Statistical analyses were performed using the statistics program SPSS (IBM, Armonk, NY, USA) for Windows (Microsoft, Redmond, WA, USA). Inferential statistics are used in an exploratory manner for generating new hypotheses. According to Fisher’s approach, *p*-values are interpreted as a metric measure of evidence against the respective null hypothesis. Thus, neither global nor local significance levels were determined, and no adjustment for multiplicity was applied. However, *p*-values < 0.05 were considered statistically significant. In addition to *p*-values, risk estimators (odds ratios [OR] or hazard ratios [HR]) and corresponding 95% confidence intervals (95% Cl) were calculated, providing additional descriptive information on the probabilities of an event under certain exposures.

For describing the data, standard univariate statistical analyses were applied. Categorical variables are shown as absolute and relative frequencies. Continuous variables are shown as median [range: minimum–maximum]. Acute toxicity was analyzed as a binary variable (grade 1–4/grade 0). Fisher’s exact test was used to quantify the association between acute toxicity and binary categorical variables (sex, TBI, any chemotherapy, CTX before RT, CTX simultaneously with RT, CTX after RT, pre-existing eye impairment, protons). The nonparametric Mann–Whitney test was used to evaluate the influence of continuous variables, such as age and Dmax irradiation dose to the eye.

Since for the observation of late toxicity no fixed time interval was defined, the time until late toxicity (grade 1–4) was analyzed using time-to-event methods, such as Kaplan–Meier estimates, log-rank tests, and univariate Cox regressions.

Finally, variables with statistically significant influence in univariate analysis (+chemotherapy pre/simultaneous/post) were investigated in a multivariate statistical analysis, enabling the evaluation of a connection and dependencies between various variables. For the analysis of acute toxicity, multivariate logistic regression was performed and a Cox regression for late toxicity.

## Results

From 2001 to 2016, 593 patients were included in this study who received a radiation dose to the eye (34.4%) and who were documented within the RiSK database. The basic characteristics of the patients, diagnosis, and treatment are shown in Table 2. 145 forms regarding pre-existing eye impairment were available. These include, for example, loss of vision, ptosis, eye muscle paresis, and strabismus. The median age of the patients was 8.48 years and the majority of patients received a radiation dose of more than 50 Gy to the eye (*n* = 270).

### Acute eye toxicity

Acute toxicity regarding the eye was documented in 435 patients (73.4%, Table 2), whereof *n* = 367 had no (grade 0) reaction or grade 1, 2, or 4 in 49, 17, and 2 patients, respectively. The majority of patients received more than 50 Gy maximum dose to the eyes (52.4%). The basic data of the patients are shown in Table 2.

Univariate analysis revealed a statistically significant association between the radiation dose > 50 Gy to the eyes and acute toxicities, with an odds ratio of 1.97 (95% CI 1.14–3.38; *p*-value 0.017). The patients who developed acute toxicities were also statistically significantly more likely to have had a pre-existing eye impairment (for example, double vision, decreased vision, ptosis, nystagmus), chemotherapy before undergoing irradiation, or radiation with protons (Table 3). About one third of the patients with documented acute toxicity were irradiated with protons (33.8%). The median age of 6.5 years (range 0.77–18.4 years) of these patients is below the average age of all patients in the study. 77.6% of the proton irradiations were done with a maximum radiation dose to the eye (Dmax) > 50 Gy and the median Dmax was 54.50 Gy (compare median Dmax of 41.0 Gy in patients without proton irradiation). The diagnosis of rhabdomyosarcoma was statistically associated with acute toxicity in the analysis (*p*-value < 0.001, odds ratio 6.34 with 95% CI 3.62–11.11). The age group of 0–4 years had an increased risk of developing acute toxicity after irradiation. Other age groups (compare Table 2) show no statistically significant relationship.

**Table 3:** Univariate influence on acute toxicity

	Toxicity grade 0 (%)	Toxicity grade 1–4 (%)	Odds ratio	95% confidence limits	*p*-value
*Total*	367 (84.4)	68 (15.6)	–	–	–
*Pre-existing eye impairment*					
Yes	84 (69.4)	37 (30.6)	4.02	2.35–6.87	< 0.001
No	283 (90.1)	31 (9.9)			
*Direct influence on vision due to tumor site*					
Yes	149 (74.9)	50 (25.1)	4.06	2.28–7.24	< 0.001
No	218 (92.4)	18 (7.6)			
*Rhabdomyosarcoma*					
Yes	50 (59.5)	34 (40.5)	6.34	3.62–11.11	< 0.001
No	317 (90.3)	34 (9.7)			
*Protons*					
Yes	109 (74.2)	38 (25.9)	2.99	1.77–5.09	< 0.001
No	258 (89.6)	30 (10.4)			
*CTx pre*					
Yes	252 (81.6)	57 (18.5)	2.37	1.20–4.68	0.007
No	115 (91.3)	11 (8.7)			
*CTx simultaneous*					
Yes	159 (81.1)	37 (18.9)	1.56	0.928–2.626	0.060
No	208 (87.0)	31 (13.0)			
*CTx post*					
Yes	154 (82.8)	32 (17.2)	1.23	0.73–2.07	0.258
No	213 (85.5)	36 (14.5)			
*CTx all*					
Yes	301 (83.8)	58 (16.2)	1.27	0.62–2.62	0.323
No	66 (86.8)	10 (13.2)			
*Age 0–4 years*					
Yes	87 (77.7)	25 (22.3)	1.87	1.08–3.24	0.034
No	280 (86.7)	43 (13.3)			
*Dmax >* *40.0–50* *Gy (group 6)*					
Yes	40 (76.9)	12 (23.1)	1.75	0.87–3.54	0.151
No	327 (85.4)	56 (14.6)			
*Dmax >* *50* *Gy (group 7)*					
Yes	87 (77.7)	25 (22.3)	1.97	1.14–3.38	0.017
No	280 (86.7)	43 (13.3)			

Categorial variables are reported as absolute and relative frequencies. Influence is reported as odds ratios and 95% confidence limits. *P*-values are from Fisher’s exact test

*CTx* chemotherapy, *Dmax* maximum dose

Additional multivariate analysis was performed for acute toxicity considering the impact of several variables (listed in Table 5). The significant influence of a pre-existing eye impairment (OR 3.47 [95% CI 1.81–6.65], *p*-value < 0.001), the diagnosis rhabdomyosarcoma (OR 5.26 [95% CI 2.47–11.23], *p*-value < 0.001), and a tumor with direct influence on the vision or the eye due its localization (OR 3.77 [95% CI 1.92–7.40], *p*-value < 0.001), i.e., tumor location on the visual apparatus (directly on the eye, chiasma region, or posterior visual tract) and thus possible tumor-related toxicity to the eye, was also confirmed in the multivariate analysis. The class of Dmax irradiation dose to the eyes and age group were not included in the multivariate analysis due to univariate logistic regression not yielding a statistically usable result, the reason being an unequal distribution within the groups. In univariate analysis, Dmax > 50 Gy and > 40.0–50 Gy were considered as single categorial variables (see Table 3). Further results can be seen in Tables 3 and 5.

### Late eye toxicity

In 268 patients (45.2%), late toxicity after radiotherapy regarding the eye was observed (Table 2). The median time to the last documented follow-up in registry was 27.04 months (range 2.2–194.0 months). A maximum late toxicity ≥ 0 occurred in 268 patients, of whom *n* = 226 showed grade 0 toxicity (grade 1 = 15, grade 2 = 11, grade 3 = 11). Five patients developed the highest degree of late toxicity (grade 4 = 5). Additional basic data of patients are shown in Table 2.

As described above, the influence of the different variables on development of late toxicity was determined by means of a survival analysis (Table 4). Within univariate analysis, a significant influence of previously occurring acute toxicity was identified (HR 7.53 [95% CI 4.01–14.15], *p*-value < 0.001). 21 patients experienced late effects after already having suffered from acute toxicity. In addition, patients diagnosed with rhabdomyosarcoma had a significantly higher risk of developing late complications after radiation (HR 3.21 [1.73–5.97], *p*-value < 0.001). Furthermore, simultaneous chemotherapy, a tumor with direct influence on the vision or the eye due its localization, and proton irradiation were significantly correlated with the risk of late toxicity (Table 4). As in patients with acute toxicity, one third of the children (33.2%) with documented late toxicity were irradiated with protons. The median age of 6.8 years was also lower than the general study average. 85.4% of the children were irradiated with a radiation dose > 50 Gy to the eye and the median Dmax was 55.4 Gy, which is clearly higher than in the children irradiated without protons (median Dmax 29.22 Gy). Children 0–4 years of age were particularly at risk for late sequelae of the eye, confirming the survival analysis with the HR of 5.04 (95% CI 1.15–22.07) and *p*-value 0.032. An analysis of the maximum irradiation dose to the eye (Dmax) proved to be difficult due to the lack of documentation in some groups of children concerning late toxicities. But a consideration of group 7 (Dmax > 50 Gy) as a single variable showed a significant influence in univariate analysis (HR 2.92 [95%-CI 1.51–5.65], *p*-value < 0.001).

**Table 4:** Univariate influence on late toxicity

	Toxicity grade 0 (%)	Toxicity grade 1–4 (%)	Hazard ratios	95% confidence limits	*p*-value
*Total*	226	42	–	–	–
*Pre-existing eye impairment*					
Yes	60 (82.2)	13 (17.8)	1.66	0.85–3.23	0.132
No	167 (85.6)	28 (14.4)			
*Direct influence on vision due to tumor seat*					
Yes	90 (75.6)	29 (24.4)	3.11	1.58–6.13	< 0.001
No	2 (100)	0 (0)			
*Rhabdomyosarcoma*					
Yes	37 (63.8)	21 (36.2)	3.21	1.73–5.97	< 0.001
No	190 (90.5)	20 (9.5)			
*Protons*					
Yes	69 (77.5)	20 (22.5)	4.45	2.30–8.63	< 0.001
No	158 (88.3)	21 (11.7)			
*TBI*					
Yes	26 (83.9)	5 (16.1)	1.18	0.46–3.03	0.727
No	201 (84.8)	36 (15.2)			
*Acute toxicity*					
Yes	22 (51.2)	21 (48.8)	7.53	4.01–14.15	< 0.001
No	205 (91.1)	20 (8.9)			
*CTx pre*					
Yes	157 (82.2)	34 (17.8)	1.84	0.81–4.19	0.139
No	70 (90.9)	7 (9.1)			
*CTx simultaneous*					
Yes	85 (80.2)	21 (19.8)	1.99	1.06–3.76	0.029
No	142 (87.7)	20 (12.3)			
*CTx post*					
Yes	88 (79.5)	18 (20.5)	1.14	0.61–2.13	0.690
No	139 (85.8)	23 (22.5)			
*CTx all*					
Yes	172 (82.7)	36 (17.3)	2.05	0.79–5.30	0.129
No	55 (91.7)	5 (9.3)			
*Age 0–4 years*					
Yes	50 (76.9)	15 (25)	5.04	1.15–22.07	0.032
No	177 (87.2)	26 (12.8)			
*Dmax 0.0–2.0* *Gy (group 1)*					
Yes	7 (3.1)	0 (0.0)	n.s.r.	n.s.r.	n.s.r.
No	219 (96.9)	42 (100.0)			
*Dmax >* *2.0–5.0* *Gy (group 2)*					
Yes	4 (1.8)	0 (0.0)	n.s.r.	n.s.r.	n.s.r.
No	222 (98.2)	42 (100.0)			
*Dmax >* *5.0–12.0* *Gy (group 3)*					
Yes	46 (20.4)	6 (14.3)	n.s.r.	n.s.r.	n.s.r.
No	180 (79.6)	36 (85.7)			
*Dmax >* *12.0–20.0* *Gy (group 4)*					
Yes	10 (4.4)	2 (4.8)	n.s.r.	n.s.r.	n.s.r.
No	216 (95.6)	40 (95.2)			
*Dmax >* *20.0–40.0* *Gy (group 5)*					
Yes	29 (12.8)	3 (7.1)	n.s.r.	n.s.r.	n.s.r.
No	197 (87.2)	39 (92.9)			
*Dmax >* *40.0–50.0* *Gy (group 6)*					
Yes	22 (9.7)	5 (11.9)	n.s.r.	n.s.r.	n.s.r.
No	204 (90.3)	37 (88.1)			
*Dmax >* *50* *Gy (group 7)*					
Yes	106 (81)	26 (61.9)	2.92	1.51–5.65	< 0.001
No	121 (88.3)	16 (38.1)			

Categorial variables are reported as absolute and relative frequencies. Influence on late toxicity is reported as hazard ratios and 95% confidence limits (from Cox regression). *P*-values are from log-rank test

*CTx* chemotherapy, *TBI* total body irradiation, *n.s.r.* no statistical result

**Fig. 1:**
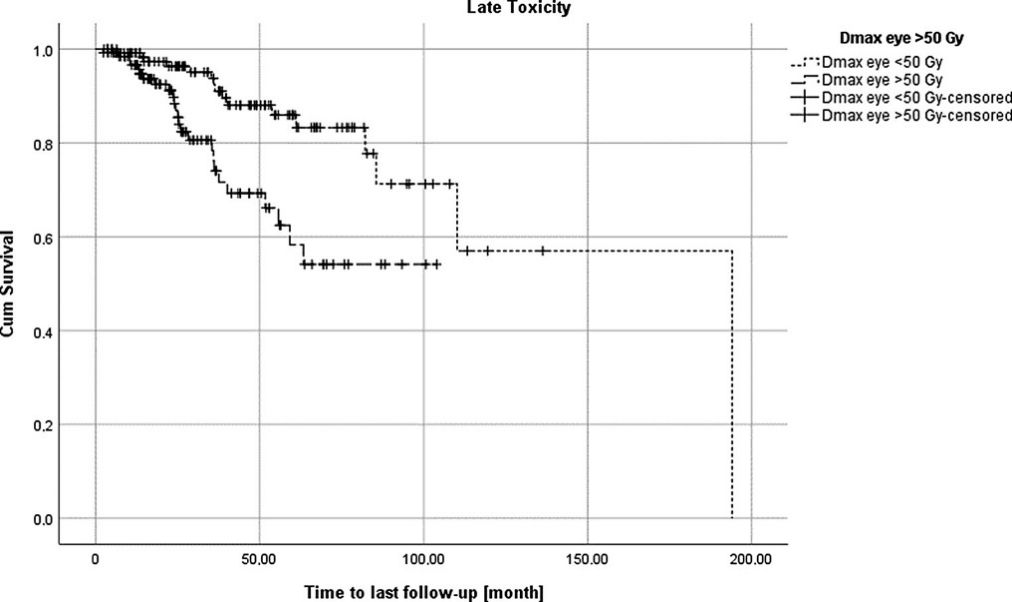
Kaplan–Meier plot of late toxicity of patients with Dmax > 50 Gy in the eye

As described in the statistical methods section, multivariate analysis of the influencing factors was subsequently performed (Table 5). The association with acute toxicity was also confirmed in multivariate analysis (HR 4.29 [95% CI 1.91–9.62], *p*-value < 0.001). The risk of developing late toxicity after irradiation with more than 50 Gy (compare Fig. 1) was significantly increased, as shown by the *p*-value 0.038 (HR 2.31 [95% CI 1.05–5.10]). Other variables, such as the diagnosis of rhabdomyosarcoma, proton therapy, or a pre-existing eye impairment, did not show any significant influence on the development of late toxicities affecting the eye within this multivariate analysis (compare Table 5).

**Table 5:** Results of the multivariable analysis of acute and late toxicity

	Acute toxicity grade 1–4				Late toxicity grade 1–4		
	Odds ratio	95% confidence interval	*p*-value		Hazard ratio	95% confidence interval	*p*-value
*Pre-existing eye impairment*	3.47	1.81–6.65	< 0.001	*Pre-existing eye impairment*	0.73	0.35–1.54	0.404
*Direct influence on vision due to tumor seats*	3.77	1.92–7.40	< 0.001	*Direct influence on vision due to tumor seats*	2.01	0.90–4.46	0.087
*Rhabdomyosarcoma*	5.26	2.47–11.23	< 0.001	*Rhabdomyosarcoma*	1.48	0.62–3.53	0.375
*Protons*	1.01	0.51–2.02	0.975	*Protons*	1.11	0.44–2.82	0.822
*CTx pre*	6.99	0.85–57.57	0.071	*CTx sim*	0.98	0.44–2.18	0.958
*TBI*	1.47	0.28–7.57	0.071	*Dmax >* *50* *Gy*	2.31	1.05–5.10	0.038
				*Acute toxicity*	4.29	1.91–9.62	< 0.001

Logistic regression was performed for the dependent binary variable acute toxicity (grade 1–4 vs. grade 0). Cox regression was performed for the time until the maximal late toxicity (grade 1–4).

*CTx* chemotherapy,* TBI* total body irradiation

The patient records of the five patients with grade 4 toxicity were casuistically reviewed to demonstrate more precise information on the individual high-grade sequelae of radiation exposure. One patient showed radiation-induced optic atrophy with visual deterioration 1 year after the end of irradiation. In another patient, opticus atrophy worsened after irradiation, so that a deterioration of vision was also described. Another child developed a severe sicca syndrome as a result of radiation. More detailed information on the course of the disease and examination reports from ophthalmologists are only partially available to the registry in the respective patient files. However, it can be stated that all three patients were irradiated with a Dmax > 50 Gy in the eye. One patient also corresponds to the age group 0–4 years, which in univariate analysis shows an increased risk of developing late toxicity in the eye.

Regrettably, for the other two patients, we had inaccurate and contradictory data regarding the demarcation between pre-therapeutic visual impairment and treatment-associated damage, which makes further consideration impossible. The cases demonstrate the significant dose dependence of the development of late toxicities. In addition, they also show the diversity of causes of late toxicity in detail (optic atrophy, sicca syndrome etc.), which cannot be further analyzed from the statistics and classification alone.

## Discussion

The eye, mainly the lens, is a particularly radiation-sensitive organ and even low doses of radiation increase the risk for subsequent ocular disorders [7, 9]. Especially the effect of radiation on the lens with regard to development of cataract has been investigated in numerous studies [7–9, 14–16]. Whelan et al. indicated in their large cohort study of the Childhood Cancer Survivor Study (CCSS) that survivors have a significantly higher risk for all ocular damages [7]. Acute toxicity reactions predominantly affect the ocular anterior segment and can cause reactions such as conjunctivitis, keratitis, or blepharitis [17]. Acute toxicities (≥ grade 1) did occur in our study in 11.5% of the patients. When analyzing the irradiation dose, the univariate analysis shows that a maximum radiation dose > 50 Gy significantly increased the risk of developing acute toxicity. In other studies the risk was already increased at appreciably lower irradiation doses. According to the ICRP (International Commission on Radiological Protection), ocular pathologies other than lens opacity after acute or fractional exposures already occurred at radiation doses of 5–20 Gy [15]. It can be assumed that the risk of an acute toxicity reaction is also increased by radiation doses lower than 50 Gy. Considering that in our case, only very few patients with documented toxicity were documented in Dmax 0–2.0 Gy, > 2.0–5.0 Gy, > 5.0–12.0 Gy, > 12.0–20.0 Gy, and > 20.0–40.0 Gy, statistical evaluation is not feasible. Acute toxicity reactions are usually reversible. For example, Gordan et al. described the self-limitation of acute complications after irradiation with 2–2.5 Gy to about 37 Gy in the treatment of orbital or conjunctival lymphomas [14]. Despite the frequent regression of acute reactions, the occurrence of toxicities and particularly severe chronic toxicities should not be underestimated—as our study impressively shows. As already demonstrated in another RiSK-based study, high-grade acute toxicities are a risk factor for developing chronic toxicity reactions [18]. In our study, the presence of acute toxicity was also significantly associated with late sequelae (*p*-value < 0.001). Thus, it is an important conclusion that an acute toxicity reaction should be avoided if possible, in order to consequently minimize the risk of severe late sequelae.

In our study, late toxicity (grade 1–4) was determined in 7.08% of patients, of whom five showed grade 4 toxicity according to the RTOG criteria (Table 1). According to Barabino et al., late reactions are mainly due to permanent vascular damage and the resulting ischemia of ocular structures, which can lead to retinopathies, cataracts, or neuropathies (these sequelae have also been observed in other studies [19]) [17]. In their study, they summarized the effects of radiation on the individual orbital structures based on other studies and evaluated them according to a radiation dose relationship. The pathophysiology of irradiation-induced cataracts is additionally based on DNA damage of lens epithelial cells and the development of posterior subcapsular opacification by migration of abnormal cells [8, 14]. The publication of Whelan et al. from the CCSS examined six different kinds of ocular sequelae originating from an irradiation dose relationship [7]. The development of dry eyes, double vision, legal blindness, cataract, retinal conditions, and glaucoma was evaluated. The occurrence of eye sequelae was compared between survivors and a sibling control group. Patients answered specific questions about the occurrence of the six diagnoses in a self-questionnaire, repeated at 5 and 10 years after diagnosis. The analysis revealed an increased risk for survivors of developing a cataract, glaucoma, legal blindness, double vision, and dry eyes compared to siblings. The relationship between the irradiation dose to the eye and the risk of late complications also showed a defined correlation: a dose of 2–5 Gy significantly increased the risk of double vision and cataract. With a radiation dose of 5–12 Gy, a significant influence on the development of dry eyes and blindness was found. Retinal conditions were more frequent at doses of 20–40 Gy or more. It should be noted that the risk of all diseases increased with higher radiation doses (also observed specifically for cataract in Tinkle et al. [16]). Unfortunately, an exact evaluation of the influence of the irradiation dose to the eye on development of late toxicity was not possible in our study, as the number of patients in the different dose groups was insufficient. However, in the multivariate analysis of the single variable Dmax > 50 Gy, the survival analysis showed significance with a *p*-value 0.038. Of the 42 patients with documented late toxicity ≥ grade 1, 25 received a radiation dose of > 50 Gy to the eye. We conclude that the risk of toxicity is lower for radiation doses below 50 Gy than above 50 Gy. An irradiation dose of 50 Gy to the eye can be observed as a critical threshold [17]. Although the context of the study by Jazmati et al. is different due to the palliative situation of the irradiated children, no late toxicities were documented in this study with a median irradiation dose of 36 Gy [20]. Another limitation of our study when compared to other studies was the inability to examine the individual specific secondary disorder. The RiSK uses the graduation of consequential damage according to RTOG/EORTC; therefore, a differentiation of specific diseases is not available [6]. The same degree can be assigned by different ocular diseases and their severity (see Table 1). An advantage of this approach can be observed. Radiation-induced damage to one structure can additionally negatively affect other surrounding ocular structures [14]. The indicated degree of toxicity is able to offer an overall impression of the acute and late sequelae, in addition to an overall assessment of the eye and vision.

The influence of chemotherapy as a risk factor for toxicity after RT has been investigated in several studies [7, 9, 11–13]. Cytostatic drugs such as platinum, vinca alkaloids, taxane, methotrexate, or even 5‑flourouracil can come with ocular toxic side effects [21, 22]. For example, vincristine, frequently used in chemotherapy of various malignant diseases, can cause diplopia, ptosis, optic atrophy, or transient cortical blindness [21, 22]. Our study also showed a significant relationship between chemotherapy and toxicities. In acute toxicity, chemotherapy prior to radiation showed notably significant effects, as did simultaneous chemotherapy for late secondary damage. The relationship between simultaneous chemotherapy and high-grade toxicities has already been suggested by Pixberg et al. [18].

Rhabdomyosarcoma was the most common diagnosis of the included patients in this study and represented a statistically significant risk factor for the development of acute (in uni- and multivariate analysis) and late toxicity (in univariate analysis). In the publication of Eade et al., rhabdomyosarcoma is mentioned as the most frequent primary tumor of the orbit in childhood [23]. Main late complications after treatment are cataract and keratopathy. Other studies also proved ocular late effects after rhabdomyosarcoma therapy in childhood and adolescence [23, 24]. The relevant pre-existing eye impairment also increased the risk for toxicity—in case of acute toxicity even significantly in uni- and multivariate analysis. It cannot be derived from the RiSK data whether previous damage to the eye was caused by the tumor, although it can be assumed. An ophthalmological examination ahead of commencing irradiation can indicate an existing damage, which can be prone to exacerbation [17]. This may help to predict or even prevent a possible risk of secondary late effects and aggravation of existing ocular restrictions.

Children aged 0–4 years had a significantly higher risk of developing acute or late toxicity in univariate analyses. Most ocular structures develop in early childhood [25]. For example, lens thickness decreases and the individual compartments change their depth and length. Due to this early childhood development, it can be assumed that irradiation at this age has an influence on development and the eye is particularly susceptible to secondary damage. The significance in our statistical evaluation can be attributed to this influence. The analysis of the influence of proton irradiation must be viewed critically with regard to any possible impact on toxicity. Even when the univariate analysis of acute toxicity and late toxicity suggests a significant influence, this finding could not be confirmed in multivariate analysis. Most probably, the observed effect was caused by a selection bias. With proton therapy, particularly very young patients diagnosed with rhabdomyosarcoma receiving relatively high radiation doses were treated. Accordingly, our descriptive evaluations show a significantly higher median irradiation dose to the eye with protons when compared to photons. A high median radiation dose for proton irradiation can also be observed in other studies such as Doyen et al. (median total prescribed dose 55.8 Gy) [26]. Therefore, the use of protons cannot be associated with any increased risk for toxicity within this study.

The patients with the highest degree of late eye toxicity were casuistically evaluated. But also the consequential damages, which were classified with grade 2 or 3, represent a major limitation in the quality of life of the affected patients. A descriptive evaluation of this collective (*n* = 22) shows a median age of 4.5 years and an average Dmax of 50.5 Gy (13 of the 22 patients belong to Dmax group 7). Also interesting is the fact that 59.1% had already documented acute damage to the eye after irradiation.

In radiotherapy, the technical possibilities are constantly being developed with the aim of minimizing acute and late sequelae. Techniques such as IMRT or tomotherapy make it possible to irradiate pathological tissue in a more conformal manner while sparing healthy surrounding tissue [27, 28]. It is well known that in addition to the radiation dose used, the volume irradiated has a major impact on organs at risk (OARs) and this is minimized by new radiation methods [29]. Maximum doses are often determined for OARs to minimize sequelae (for example, < 55 Gy at the base of the skull, optic chiasm, optic nerve) [26]. Nevertheless, studies show that late effects occur even with these techniques [28]. In 2020, Bishr et al. show that radiation therapy is used very commonly in pediatric cancer treatment—both curatively and palliatively [30]. Children with radiation-related late effects are forced to live with these effects for significantly more years, and this can have a significant impact on quality of life [28]. This is why, in our view, it is so important to minimize and ideally prevent radiation damage in childhood.

There are limitations of our study. Unfortunately, we did not have all information on late toxicity for our cohort and FU time was still limited. Moreover, information on specific ocular disorders following radiotherapy was not given. Finally, when considering all subgroups and techniques used, the number of patients was still restricted. Data were not analyzed in a matched-pair analysis. Furthermore, we did not investigate consecutive interventions or therapies for eye disorders, which would be of interest to understand the impact of any specific disorder and to better define the role of RT in the future. To conclude, an irradiation dose of > 50 Gy to the eye represented a significantly increased risk for toxicity of the eye. However, young age and RMS diagnosis showed a significant influence as well. To our knowledge, RiSK was the only prospective registry for recording acute and late effects of radiotherapy in childhood and adolescence. The documentation was carried out by qualified radio-oncologists and based on standardized medical follow-up examinations, which is crucial for the aftercare of specific side effects [31]. Therefore, the data from the RiSK must be considered objective with regard to eye damage. Other studies dealing with the same problem are analyzed on the basis of information from patient self-interviews, which are subjective views of the patients [7, 9].

## Conclusion

The eye is very sensitive to radiation and, especially in early childhood, any exposure to radiation can have an effect on the development of the eye and the occurrence of secondary damage. Our study indicates that different factors have an influence on the development of eye toxicity after irradiation. Acute and late toxicity seem to be correlated with a high radiation dose to the eye (> 50 Gy). Also, patients having treatment for a rhabdomyosarcoma diagnosis seem to be particularly prone to developing eye disorders. However, even below a dose of 50 Gy is there a certain risk for severe acute and especially chronic toxicities. Therefore, radiation doses to the eye should generally be kept as low as possible to reduce the risk of toxicity. Any radiation therapy in a child and in the craniofacial area has to be carefully balanced against the risk for life-long sequelae.

## References

[1] Willich N, Ernst I, Pape H (2009). Evaluation of side effects after radiotherapy in childhood and adolescence: from retrospective case reports to a prospective, multicentric and transnational approach. Strahlenther Onkol.

[2] Willich N, Schuck A, Bölling T (2005) Studienprotokoll RiSK. https://www.ukm.de/fileadmin/ukminternet/daten/kliniken/strahlentherapie/dokumente/RiSK/Studienprotokoll_RiSK.pdf. Accessed 10 Mar 2020

[3] Mansouri I, Allodji RS, Hill C (2019). The role of irradiated heart and left ventricular volumes in heart failure occurrence after childhood cancer. Eur J Heart Fail.

[4] Scharl S, Combs SE (2019). Endocrine deficiency after radiotherapy of brain tumors in children and young adults. Strahlenther Onkol.

[5] Sauer R (2010). Strahlentherapie und Onkologie.

[6] Cox JD, Stetz J, Pajak TF (1995). Toxicity criteria of the Radiation Therapy Oncology Group (RTOG) and the European Organization for Research and Treatment of Cancer (EORTC). Int J Radiat Oncol Biol Phys.

[7] Whelan KF, Stratton K, Kawashima T (2010). Ocular late effects in childhood and adolescent cancer survivors: a report from the childhood cancer survivor study. Pediatr Blood Cancer.

[8] Wilde G, Sjöstrand J (1997). A clinical study of radiation cataract formation in adult life following gamma irradiation of the lens in early childhood. Br J Ophthalmol.

[9] Chodick G, Sigurdson AJ, Kleinerman RA (2016). The risk of cataract among survivors of childhood and adolescent cancer: a report from the childhood cancer survivor study. Radiat Res.

[10] Whelan RJ, Saccomano B, King R (2018). Radiation-induced cataracts in children with brain tumors receiving craniospinal irradiation. J Pediatr Hematol Oncol.

[11] Bölling T, Geisenheiser A, Pape H (2011). Hypothyroidism after head-and-neck radiotherapy in children and adolescents: preliminary results of the “Registry for the Evaluation of Side Effects After Radiotherapy in Childhood and Adolescence” (RiSK). Int J Radiat Oncol Biol Phys.

[12] Stoppel G, Eich HT, Matuschek C (2017). Lung toxicity after radiation in childhood: results of the International Project on Prospective Analysis of Radiotoxicity in Childhood and Adolescence. Radiother Oncol.

[13] Rösler P, Christiansen H, Kortmann RD (2015). Hepatotoxicity after liver irradiation in children and adolescents: results from the RiSK. Strahlenther Onkol.

[14] Gordon KB, Char DH, Sagerman RH (1995). Late effects of radiation on the eye and ocular adnexa. Int J Radiat Oncol Biol Phys.

[15] Hamada N, Azizova TV, Little MP (2019). An update on effects of ionizing radiation exposure on the eye. Br J Radiol.

[16] Tinkle CL, Pappo A, Wu J (2020). Efficacy and safety of limited-margin conformal radiation therapy for pediatric rhabdomyosarcoma: long-term results of a phase 2 study. Int J Radiat Oncol Biol Phys.

[17] Barabino S, Raghavan A, Loeffler J (2005). Radiotherapy-induced ocular surface disease. Cornea.

[18] Pixberg C, Koch R, Eich HT (2016). Acute toxicity grade 3 and 4 after irradiation in children and adolescents: results from the IPPARCA collaboration. Int J Radiat Oncol Biol Phys.

[19] Akbaba S, Foerster R, Nicolay NH (2018). Linear accelerator-based stereotactic fractionated photon radiotherapy as an eye-conserving treatment for uveal melanoma. Radiat Oncol.

[20] Jazmati D, Butzer S, Hero B (2020). Long-term follow-up of children with neuroblastoma receiving radiotherapy to metastatic lesions within the German Neuroblastoma Trials NB97 and NB 2004. Strahlenther Onkol.

[21] Armstrong C, Sun LR (2020). Neurological complications of pediatric cancer. Cancer Metastasis Rev.

[22] Schmoll H, Höffken K, Possinger K (2006). Kompendium Internistische Onkologie Standards in Diagnostik und Therapie.

[23] Eade E, Tumuluri K, Do H (2017). Visual outcomes and late complications in paediatric orbital rhabdomyosarcoma. Clin Exp Ophthalmol.

[24] Heyn R, Ragab A, Raney RB (1986). Late effects of therapy in orbital rhabdomyosarcoma in children. A report from the Intergroup Rhabdomyosarcoma Study. Cancer.

[25] Mutti DO, Sinnott LT, Lynn Mitchell G (2018). Ocular component development during infancy and early childhood. Optom Vis Sci.

[26] Doyen J, Jazmati D, Geismar D (2019). Outcome and patterns of relapse in childhood parameningeal rhabdomyosarcoma treated with proton beam therapy. Int J Radiat Oncol Biol Phys.

[27] Salfelder MA, Kessel KA, Thiel U (2020). Prospective evaluation of multitarget treatment of pediatric patients with helical intensity-modulated radiotherapy. Strahlenther Onkol.

[28] Lockney NA, Friedman DN, Wexler LH (2016). Late toxicities of intensity-modulated radiation therapy for head and neck rhabdomyosarcoma. Pediatr Blood Cancer.

[29] Lundgaard AY, Hjalgrim LL, Rechner LA (2020). The risk of late effects following pediatric and adult radiotherapy regimens in Hodgkin lymphoma. Strahlenther Onkol.

[30] Bishr MK, Zaghloul MS, Elmaraghi C (2020). The radiotherapy utilization rate in pediatric tumors: an analysis of 13,305 patients. Radiother Oncol.

[31] Jouin A, Helfre S, Bolle S (2019). Adapted strategy to tumor response in childhood nasopharyngeal carcinoma: the French experience. Strahlenther Onkol.

